# Anastomotic Leak After Esophagectomy: Modern Approaches to Prevention and Diagnosis

**DOI:** 10.7759/cureus.80091

**Published:** 2025-03-05

**Authors:** Andrei I Gritsiuta, Christopher J Esper, Kavita Parikh, Sreeram Parupudi, Roman V Petrov

**Affiliations:** 1 Department of Surgery, University of Pittsburgh Medical Center, Pittsburgh, USA; 2 Department of Cardiothoracic Surgery, University of Texas Medical Branch, Galveston, USA; 3 Department of Gastroenterology and Hepatology, University of Texas Medical Branch, Galveston, USA

**Keywords:** anastomotic leak(al), esophageal cancer (ec), minimally invasive esophagectomy (mie), post-esophagectomy, vacuum assisted closure (vac)

## Abstract

Anastomotic leak (AL) remains one of the most serious complications following esophagectomy, contributing to significant morbidity, prolonged hospital stays, and increased mortality. Despite advancements in surgical techniques and perioperative care, AL continues to challenge surgeons and negatively impact patient outcomes. Various factors contribute to its development, including patient-specific comorbidities, tumor characteristics, anastomotic technique, conduit perfusion, and perioperative management. Prevention strategies have evolved with the integration of intraoperative techniques such as fluorescence-guided perfusion assessment, omental reinforcement, and meticulous surgical handling of the gastric conduit. Emerging technologies, including endoluminal vacuum therapy (EVT) and multimodal perioperative protocols, have demonstrated potential in reducing leak incidence and improving management. Diagnosing AL remains complex due to its variable presentation, necessitating a combination of clinical evaluation, inflammatory markers, imaging studies, and endoscopic assessments. While routine postoperative imaging has shown limited sensitivity, on-demand CT and endoscopic evaluations play a crucial role in early detection and intervention. This review provides a comprehensive analysis of the risk factors, prevention strategies, and diagnostic modalities for AL after esophagectomy, incorporating recent advancements and emerging technologies.

## Introduction and background

Esophageal cancer remains a major public health concern, with an estimated 22,370 new cases and 16,130 deaths projected in the U.S. in 2025 [1]. Despite recent advancements, the five-year survival rate remains dismal at 22% [1]. However, improvements in imaging and endoscopic techniques have facilitated earlier detection, increasing the number of patients eligible for esophagectomy [2,3]. This trend underscores the critical need for effective strategies to manage complications such as anastomotic leak (AL), a major determinant of postoperative morbidity and mortality [4].

The foundation for modern esophageal surgery was laid in 1913 with Franz Torek’s pioneering esophagectomy, marking the beginning of over a century of advancements in thoracic surgery. Today, esophagectomy remains a cornerstone of treatment for esophageal cancer and other complex esophageal diseases, involving both resection and reconstruction of the upper digestive tract [3]. While crucial for long-term survival in locally advanced esophageal cancer, esophagectomy carries significant risks. AL, one of the most feared complications, leads to prolonged hospitalization, increased healthcare costs, and long-term sequelae such as stricture formation and diminished quality of life [5]. Additionally, emerging evidence suggests that AL may contribute to increased cancer recurrence, further underscoring its impact on oncologic outcomes [6].

The reported incidence of AL varies widely, with earlier studies citing rates as high as 50% and associated mortality ranging from 30% to 60% [5,7]. However, more recent data from a meta-analysis by Rahouma et al. indicate a lower AL incidence of 9.06%, with 30-day and 90-day mortality rates of 1.89% (95% CI: 1.55-2.30) and 2.43% (95% CI: 1.89-3.13), respectively, reflecting advancements in surgical techniques and perioperative care [8]. A study by Gujjuri et al. further highlighted the profound impact of AL on patient outcomes, demonstrating an overall complication rate of 91% and major complications in 11% of patients with AL, compared to significantly lower rates of 44% and 7%, respectively, in those without leaks [9].

The evolution of esophagectomy techniques-including Ivor Lewis, McKeown, transhiatal, Sweet, minimally invasive, and hybrid approaches-reflects efforts to tailor surgical strategies to tumor location, disease extent, and patient-specific factors [10]. Each approach presents distinct advantages and challenges, particularly regarding the anastomotic site [10]. Intrathoracic anastomoses generally have lower AL rates but carry a higher risk of severe complications if a leak occurs, whereas cervical anastomoses, while more accessible for intervention, exhibit higher rates of leaks and stricture formation [11]. AL presentations range from asymptomatic cases to life-threatening sepsis and mediastinitis, with prognosis heavily dependent on the degree of contamination, timing of diagnosis, and effectiveness of intervention.

Recent advancements in perioperative care have significantly improved AL management and reduced associated mortality. Innovations in critical care, nutritional optimization, and sepsis management have enhanced patient outcomes. Additionally, interventional radiology techniques, such as image-guided drainage, have minimized the need for invasive surgical interventions, while endoscopic approaches, including stent placement and vacuum-assisted closure, offer minimally invasive alternatives [12]. Intraoperative strategies such as anastomotic reinforcement with an omental flap have also demonstrated potential in reducing leak rates, highlighting the importance of a multidisciplinary approach [13].

Despite these advancements, the optimal management strategy for AL remains debated, with no universally accepted standard. Treatment approaches vary widely depending on clinical presentation and available resources. Small, localized leaks may be managed conservatively with bowel rest, intravenous antibiotics, and drainage, whereas larger or more complex leaks often necessitate endoscopic interventions, vacuum-assisted therapies, or surgical revision [14,15]. A comprehensive understanding of AL pathophysiology, coupled with individualized patient assessment, is essential for determining the most effective treatment approach.

This study aims to provide a comprehensive review of the diagnosis, prevention, and endoscopic management of ALs following esophagectomy, with a focus on contemporary strategies and recent advancements. By consolidating existing literature and clinical insights, this review explores AL pathophysiology, clinical presentation, and current therapeutic approaches, highlighting the diverse range of available interventions. The objective is to synthesize the evolving landscape of AL management, offering insights that facilitate improved patient outcomes in this challenging clinical scenario.

## Review

Definition of anastomotic leak

AL is one of the most impactful complications following esophagectomy, significantly compromising both short-term recovery and long-term survival. Historically, variations in the definition of AL have led to inconsistencies in diagnosis and reporting. It is broadly characterized as the pathological leakage of luminal contents from a surgically created connection between two hollow viscera, resulting from a full-thickness defect in the gastrointestinal tract [16]. Earlier studies often required radiological confirmation or specific clinical severity thresholds, while others included only leaks necessitating surgical intervention, excluding cases managed conservatively or with minimally invasive techniques such as endoscopic stenting or drainage [7]. The introduction of the Esophagectomy Complications Consensus Group (ECCG) definition, "a full-thickness gastrointestinal defect involving the esophagus, anastomosis, staple line, or conduit, irrespective of presentation or method of identification," has facilitated standardization in reporting and outcome evaluation across clinical centers [17].

The ECCG classification further refines AL categorization based on clinical severity and management approach, allowing for consistent benchmarking and comparative analysis. Type 1 leaks require no deviation from standard postoperative management or are managed conservatively with diet modification and/or medical therapy. Type 2 leaks necessitate non-surgical interventions such as percutaneous drainage, endoscopic therapy, or wound opening, while Type 3 leaks require surgical intervention. This framework has improved our understanding of AL incidence and clinical impact; however, variations in reporting across studies persist. A review of 39 randomized controlled trials on esophageal squamous cell cancer found that fewer than half (17/39) reported AL rates, despite its critical clinical significance [18].

Detecting AL remains a significant challenge. A retrospective study found that 49% of leaks were initially missed on postoperative esophagram, which demonstrated low sensitivity (51%) [19]. Patients with missed leaks had a higher incidence of uncontained leaks (81% vs. 29%), increased readmission rates (70% vs. 39%), and a greater likelihood of requiring reoperation (44% vs. 11%). Reported AL rates range from 11.4% to 12.5% following esophagectomy, with associated 30-day and 90-day mortality rates of approximately 2.0% and 4.5%, respectively [20].

Large multicenter studies provide further insights into AL-related mortality. An analysis of 7,595 esophagectomies from the Society of Thoracic Surgeons Database demonstrated significantly higher mortality among patients requiring surgical intervention for AL (11.6%; 38 of 327) compared to those managed medically (4.4%; 20 of 458; P < 0.001) [21]. Another study of 559 patients undergoing intrathoracic anastomosis found that 7.9% experienced AL within 30 days, with a subsequent 90-day mortality rate of 18.2%, compared to 6.2% in those without AL (P = 0.003). AL was linked to a threefold increase in postoperative mortality risk (adjusted OR 3.0, 95% CI: 1.2-7.2) [22]. Similarly, an analysis of 2,944 esophagectomy patients across 30 centers reported a 30-day mortality rate of 5.0% and an in-hospital mortality rate of 7.3%. Pulmonary complications (38.1%) and AL (10.2%) were leading causes of postoperative mortality, with AL accounting for 19.1% of all deaths and being over four times more prevalent among patients who died (36.1% vs. 8.8%) [23].

The long-term implications of AL are profound. A study of 915 patients with a mean follow-up of 30.8 months found that postoperative complications occurred in 59.2% of cases, with 50.2% classified as Clavien-Dindo (CD) grade 3 or higher (i.e., requiring intervention, causing organ dysfunction, or resulting in death). AL was identified in 14.8% of patients, with 62.2% classified as CD grade 3 or higher. Multivariable analysis demonstrated that AL significantly reduced long-term survival (HR: 1.68; 95% CI: 1.25-2.24), with an even greater impact for CD grade 3 or higher leaks (HR: 1.83; 95% CI: 1.30-2.58). Notably, other postoperative complications were not significantly associated with long-term survival, emphasizing the uniquely detrimental impact of AL [24].

A subsequent multicenter study analyzing data from 1,225 patients reinforced these findings. AL occurred in 18.4% of cases and was associated with a significantly lower five-year survival rate (44.0%) compared to those without AL (57.2%; P = 0.005) [25]. In contrast, a retrospective review of 172 minimally invasive esophagectomies found that while AL was associated with worse short-term outcomes, it did not significantly impact long-term survival, with no difference in one-year (79% vs. 83%) or five-year (50% vs. 47%) survival rates (P = 0.758) [26].

Several mechanisms may explain the adverse effects of AL on survival, including direct mortality, delayed recovery, systemic inflammation, and impaired administration of adjuvant therapies. Severe AL frequently results in prolonged hospitalization, intensive care stays, and invasive interventions, all of which can reduce physical resilience and delay critical postoperative treatments such as adjuvant therapy [14,27]. Additionally, complications such as mediastinitis and sepsis may induce systemic inflammation, potentially creating an environment that facilitates cancer recurrence [28].

The association between AL and cancer recurrence has been reported in multiple studies. A systematic review of seven studies involving 5,433 esophagectomy patients found AL rates ranging from 7.2% to 11.2%, with recurrence rates among AL patients varying from 9% to 56% [29]. Markar et al. demonstrated that severe AL was linked to a significant reduction in median overall survival (35.8 vs. 54.8 months; P = 0.002) and disease-free survival (34 vs. 47.9 months; P = 0.005) [23]. After adjusting for confounders, AL was associated with a 28% higher risk of mortality (HR: 1.28, 95% CI: 1.04-1.59; P = 0.022) and increased rates of overall recurrence (OR: 1.35, 95% CI: 1.15-1.73; P = 0.011), locoregional recurrence (OR: 1.56, 95% CI: 1.05-2.24; P = 0.030), and mixed recurrence (OR: 1.81, 95% CI: 1.20-2.71; P = 0.014) [23].

Despite advancements in standardizing definitions and classifications, AL remains a formidable challenge in esophagectomy. The ECCG classification has enhanced our understanding of AL and its clinical implications, paving the way for improved patient outcomes and more effective management strategies.

Risk factors for anastomotic leak

AL arises from a combination of factors, including patient characteristics, anatomical challenges, surgical techniques, and perioperative management. These interconnected variables create a complex risk profile that requires thorough consideration during surgical planning and patient care [30]. Widely recognized contributors to AL include patient comorbidities, the use of neoadjuvant treatments, anastomotic location, surgical technique, and perioperative monitoring [14,27,31]. Many of these factors are modifiable, presenting opportunities for tailored interventions aimed at reducing risk. However, no consensus exists on the relative importance of specific risk factors, leading to a lack of reliable predictive models and standardized tools for preoperative risk assessment. This variability underscores the complexity of mitigating AL and highlights the need for further research to develop comprehensive and validated risk assessment strategies.

Anatomical and Physiological Challenges

The intrinsic structure of the esophagus plays a significant role in its susceptibility to complications. Unlike other segments of the gastrointestinal tract, it lacks a serosal layer, relying solely on its adventitia for structural support. This absence compromises tensile strength and diminishes the esophagus's ability to withstand mechanical and ischemic stress during healing [32]. These structural limitations, combined with the unique physiological dynamics of the thoracic cavity, make esophageal anastomoses particularly prone to complications.

Intrathoracic anastomoses face specific challenges due to the physiological environment of the thoracic cavity. Negative pressure during respiration may place mechanical strain on the anastomosis, while ventilatory dynamics, barometric fluctuations, and lung compression exacerbate the risk of complications. The esophagogastric conduit, relocated into the thoracic cavity, is exposed to novel physiological forces. Supradiaphragmatic mechanics and lung expansion exert pressure on the conduit, while negative thoracic pressure facilitates retrograde aspiration of gastric contents and bacteria, increasing the risk of impaired healing and infection [33]. Hence, the Ivor Lewis esophagectomy is associated with a unique risk profile for AL [14].

The gastric conduit relies on a tenuous blood supply from a single vessel, which is distant from the anastomotic site, making it vulnerable to ischemia in watershed areas. Luketich et al. described a modification of minimally invasive Ivor Lewis esophagectomy (MIE-chest), involving laparoscopic gastric mobilization, thoracoscopic esophageal mobilization, and intrathoracic anastomosis through a mini-incision, with routine placement of feeding jejunostomy tubes and pyloric drainage procedures [34]. In this cohort of 530 patients, AL requiring surgery occurred in 4%. Housman et al. reported an AL rate of only 1.82% in 110 patients following a modified approach that emphasized vascular preservation, careful mobilization of the stomach, pyloroplasty, and tension-free anastomosis using an EEA (Medtronic, Minneapolis, MN) stapler [35].

Recent studies comparing intrathoracic and cervical anastomotic leaks following esophagectomy highlight significant findings. Intrathoracic leaks were associated with a higher need for interventions, such as stenting or vacuum therapy, while cervical leaks exhibited increased rates of reoperation, sepsis, and other complications. Independent predictors for both leak types included diabetes and longer operative time, with hypertension, chronic steroid use, and advanced disease stage further linked to intrathoracic leaks. Despite these differences, overall morbidity and mortality outcomes were comparable between the two leak types, challenging traditional assumptions about the severity of intrathoracic leaks [36].

Cervical anastomoses present additional challenges due to increased tension and compromised blood supply at the gastric fundus [37]. The longer conduit required for this approach increases mechanical tension at the anastomotic site and heightens the risk of vascular compromise, particularly in the gastric fundus, where perfusion is already limited. Additionally, the indications for cervical anastomoses-such as proximal tumors, higher radiation exposure, advanced pathologic stage, postoperative arrhythmia, and prior esophagogastric surgeries-further contribute to their elevated risk of leaks [38]. Cervical anastomoses have been associated with a higher AL rate compared to intrathoracic anastomoses (12.3% vs. 9.3%; P = 0.006) [21]. Despite these challenges, the choice of anastomotic site does not significantly affect overall mortality rates [14,21].

Inadequate blood supply to the gastric conduit, or conduit ischemia, is a critical factor in AL development [39]. Perfusion from the isolated right gastroepiploic artery is often insufficient to meet the metabolic demands of the entire conduit. Perfusion-related complications, including ischemia and necrosis, remain prevalent, with rates ranging from 2.5% to 20% [40].

Complete conduit necrosis, while rare, occurs in less than 3% of cases and is associated with severe clinical consequences [41]. Advances in postoperative endoscopy, coupled with a lowered threshold for its use, have led to more frequent identification of localized conduit necrosis. Although some overlap exists between the definitions of conduit necrosis and AL, stratification of these complications underscores the increasing role of endoscopic and interventional radiology techniques. Notably, surgical management for significant AL rarely requires diversion, in contrast to extensive conduit necrosis [17].

Intraoperative monitoring of conduit perfusion is essential for early detection of ischemia and optimization of anastomotic site selection (Video 1). Techniques such as angiography, transmucosal oxygen saturation measurements, and esophagogastroduodenoscopy are commonly employed [42]. Emerging optical technologies, including laser Doppler flowmetry, near-infrared spectroscopy, and fluorescence imaging with indocyanine green (ICG), provide real-time, high-resolution assessments of blood flow [43]. ICG fluorescence imaging has gained traction due to its practicality, given its short plasma half-life and biliary excretion, enabling visualization of microvascular networks and assessment of perfusion impairment [44]. Parameters such as the "90-second rule," which measures the time from ICG enhancement at the right gastroepiploic artery to the gastric tip, help guide intraoperative decisions [45]. Additionally, the distance between the perfusion demarcation point on the gastric conduit and the anastomotic site has been associated with AL risk; longer distances correspond to higher AL rates (45% vs. 2%; P < 0.0001) [39].

**Video 1:** Indocyanine green (ICG) fluorescence imaging for gastric conduit perfusion assessment. This video is from the author's own research and demonstrates the use of ICG fluorescence imaging for intraoperative assessment of gastric conduit perfusion during esophagectomy. ICG is administered intravenously and visualized using a near-infrared (NIR) fluorescence imaging system, allowing real-time identification of ischemic and well-perfused areas. This technique aids in determining the optimal anastomotic site, reducing the risk of anastomotic leaks and ischemic complications. While ICG is widely used for perfusion assessment, it is contraindicated in patients with hypersensitivity to ICG, iodine, or shellfish, necessitating alternative methods such as oxygen saturation endoscopic imaging (OXEI).

Campbell et al. retrospectively analyzed 90 esophagectomy cases and observed a significant reduction in AL rates from 20% in the first 60 patients to 0% in the subsequent 30 patients following the introduction of intraoperative ICG evaluation [46]. Ladak et al. conducted a meta-analysis of 17 studies, reporting a pooled AL rate of 10% with ICG usage [47]. Notably, the use of ICG combined with intraoperative interventions to improve perfusion reduced the risk of AL by 69% (OR: 0.31, 95% CI: 0.15-0.63). Similarly, Van Daele et al. reviewed 19 studies and found that ICG-guided evaluation of gastric graft perfusion was associated with a significantly lower AL rate (10% vs. 20.6% in the control group, P < 0.001) [48]. When poorly perfused cases were excluded, AL rates dropped to 6.3% in the well-perfused group compared to 20.5% in the control group (P < 0.001). Surgical plans altered based on ICG images achieved AL rates of 6.5%, comparable to the well-perfused group but significantly lower than the poorly perfused group (47.8%, P < 0.001) [48].

Conversely, Casas et al. analyzed 3,171 patients, including 381 who underwent ICG fluorescence imaging, and found no significant difference in AL rates between the ICG group (9%) and the no-ICG group (9%; OR: 0.85, 95% CI: 0.53-1.28, P = 0.45) [49]. This suggests that while ICG is a valuable tool, its impact may vary depending on clinical context and implementation. They hypothesized that ICG might be particularly beneficial for cervical anastomoses, where the conduit tip is often poorly perfused and under higher tension compared to intrathoracic anastomoses, which are subject to less handling and extrinsic compression [49].

Despite its advantages, fluorescence imaging remains qualitative and lacks precise quantification of blood flow. Studies have identified a flow speed of 1.76 cm/sec or less as an independent predictor of AL, determined via receiver operating characteristic analysis (P = 0.004), emphasizing the importance of intraoperative blood flow evaluation to identify high-risk patients [50]. Additionally, a promising quantitative method using time-fluorescence intensity curves, facilitated by SPY-Q (Stryker, Portage, MI) software, provides relative and absolute fluorescence intensity values [51]. This approach enables objective detection of perfusion deficits that may not be macroscopically visible, offering a valuable tool for improving surgical decision-making. Further supporting these findings, reviews of multiple studies have highlighted that time-related parameters demonstrate a superior correlation with perfusion compared to absolute intensity measures in clinical settings [52]. Notably, "time to fluorescence" has been identified as the most promising parameter for quantifying fluorescence angiography, with fluorescence-time curves enabling the derivation of various perfusion-related metrics beyond simple fluorescence intensity [53]. Collectively, these insights suggest that quantitative evaluation of blood flow, particularly through time-based parameters, may enhance the predictive accuracy of ICG fluorescence imaging. Future research should focus on standardizing these methods and integrating them into clinical workflows to improve outcomes in esophageal surgery.

Fujifilm's ELUXEO Vision System (FUJIFILM Healthcare Americas Corporation, Lexington, MA) provides real-time oxygen saturation imaging (OXEI) to assess conduit vascularization without the need for injectable dyes (Video 2). In a study of three patients undergoing robot-assisted esophagectomy, OXEI was used intraoperatively with the ELUXEO Vision 5-mm laparoscopic camera to assess gastric conduit perfusion after pull-up into the chest. Two patients had ischemic zones identified by OXEI, which were resected to optimize viability, while one patient had uniformly robust vascularity. All three had uneventful postoperative courses, with no anastomotic leaks or major complications, and were discharged within 10 days [54]. As a dye-free, real-time alternative to ICG, OXEI provides continuous perfusion monitoring and may help guide intraoperative decision-making. While promising, prospective studies are needed to further validate its efficacy compared to existing perfusion assessment methods.

**Video 2:** Real-time oxygen saturation imaging for gastric conduit perfusion using ELUXEO Vision. This video is from the author's own research and demonstrates the use of oxygen saturation endoscopic imaging (OXEI) with the ELUXEO Vision System (FUJIFILM Healthcare Americas Corporation, Lexington, MA) for intraoperative assessment of gastric conduit perfusion during esophagectomy. The 5-mm laparoscopic camera provides real-time, dye-free visualization of tissue oxygenation, allowing identification of ischemic zones and optimization of the anastomotic site. This method serves as an alternative to indocyanine green (ICG) fluorescence imaging, particularly in patients with contraindications to ICG, iodine, or shellfish. OXEI offers continuous, non-pharmacologic perfusion monitoring, enhancing intraoperative decision-making and potentially reducing complications such as anastomotic leaks and tissue necrosis.

Emerging technologies such as optical coherence tomography and sidestream darkfield microscopy may provide more detailed insights into microvascular flow and venous congestion, though further validation is required [43].

Advances in perfusion monitoring are critical for reducing ischemia-related complications. Enhanced intraoperative methodologies, combined with the adoption of preoperative ischemic conditioning, such as laparoscopic ligation or embolization of the left gastric artery, show promise in improving conduit perfusion. Ischemic preconditioning aims to enhance the gastric fundus’s tolerance to ischemia through microvascular redistribution [55,56]. While its impact on AL rates has been limited, it may reduce the severity of leaks by increasing ischemic resistance [57]. This improvement likely results from enhanced tissue adaptation rather than increased blood flow through neovascularization. Clarifying its role in AL prevention and understanding its mechanisms may help refine its application in esophageal reconstruction and improve surgical outcomes.

Patient-Specific Risk Factors

Several patient-related factors significantly impact anastomotic integrity and contribute to the risk of AL. These include malnutrition, hypoalbuminemia, advanced age, obesity (BMI >30 kg/m²), alcohol abuse, and comorbidities such as diabetes, smoking, hypertension, chronic kidney disease, chronic obstructive pulmonary disease (COPD), myocardial infarction, heart failure, and cardiac arrhythmias [21]. Atherosclerotic calcification of the celiac artery and descending aorta, systemic atherosclerosis, and impaired vascular perfusion to the gastric conduit further exacerbate ischemic risks by compromising tissue perfusion [58].

Patients with nodal involvement or advanced tumor stages face additional challenges, including compromised vascularity and increased surgical complexity, which elevate AL risk. These cases require meticulous preoperative evaluation and planning to mitigate complications. Oncological treatments, particularly neoadjuvant chemoradiotherapy (nCRT), further complicate the risk profile by impairing tissue healing and diminishing vascularity, increasing the likelihood of leaks [59]. However, the timing of surgery following nCRT plays a crucial role in mitigating these risks. A retrospective study by Roh et al. analyzing 348 esophagectomy patients found that performing esophagectomy with cervical anastomosis within 35 days of completing nCRT was associated with a significantly lower AL risk (7.3%) compared to delaying surgery beyond 35 days (20.0%; P = 0.007) or forgoing nCRT entirely (14.7%; P = 0.044) [60]. These findings highlight the importance of carefully timing surgical interventions after nCRT to optimize outcomes and minimize AL risk.

Radiation doses exceeding 31 Gy have been associated with a significant increase in AL incidence [61,62]. However, a large European multicenter study found no significant increase in AL rates with doses up to 45 Gy, a finding supported by studies reporting no elevated risk with an average dose of 41.1 Gy [63,64]. Juloori et al. conducted a retrospective analysis of 285 esophageal cancer patients treated with trimodality therapy, reporting an overall leak rate of 11%, with clinically relevant AL (grade 2 or higher) occurring in 6% of cases [65]. Notably, anastomoses constructed within the radiation field had significantly higher rates of all-grade and clinically relevant leaks (39% and 30%, respectively) compared to out-of-field anastomoses (2.6% and 1.0%; P < 0.0001) [65]. Similarly, a later study evaluating 89 esophageal cancer patients treated with trimodality therapy found a 60% overall rate of anastomotic complications, with cervical anastomoses exhibiting significantly higher rates (83%) compared to thoracic anastomoses (29%) (P < 0.01). Radiation dose to gastric substructures was not associated with anastomotic complications in either study, underscoring the critical role of surgical planning in minimizing risks and optimizing outcomes [66].

Recent national-level data further emphasize the equivalency of thoracic and cervical anastomoses in AL outcomes following nCRT. A study analyzing 908 esophagectomy patients found no significant difference in AL rates between thoracic (12%) and cervical anastomoses (14%; P = 0.4). Similarly, mortality (2% vs. 3%) and hospital length of stay (16 vs. 17 days) were comparable between the two approaches [67]. These findings highlight the importance of tailoring surgical approaches to individual patient characteristics and surgeon expertise rather than focusing solely on anastomotic location.

Anti-angiogenic therapies, such as bevacizumab, pose additional challenges by compromising vascular integrity and further complicating surgical outcomes [31]. These agents contribute to delayed healing and an increased likelihood of ischemic complications, particularly in the context of esophageal reconstruction.

Perioperative Factors

Perioperative management plays a crucial role in influencing AL risk. Blood transfusions have been identified as a significant risk factor, with higher AL rates observed in patients requiring transfusions. Simitian et al. reported a marked difference in risk between transfused patients (52.9%) and those who did not require transfusion (16.8%, P = 0.0017), potentially linked to the immunomodulatory effects of transfused blood [26]. Prolonged operative times further contribute to elevated AL rates, likely due to extended ischemic periods and heightened inflammatory responses during surgery.

Hemodynamic management is another critical determinant in perioperative care. Excessive fluid administration can lead to anastomotic edema, while overly restrictive fluid management may compromise perfusion [68]. Goal-directed therapy, using parameters such as stroke volume and mean arterial pressure (MAP), has been shown to optimize fluid balance and maintain adequate perfusion [69]. Standardized hemodynamic protocols (SHPs) have demonstrated significant benefits in improving postoperative outcomes after esophagectomy, particularly in reducing the incidence of AL and conduit necrosis. Evidence suggests that such protocols, which include restricted perioperative fluids and vasopressor use to maintain MAP >70 mmHg, can reduce complications such as pneumonia, ICU stays, and AL. The implementation of SHPs within an enhanced recovery pathway has been associated with an AL rate of 5.2% and zero conduit necrosis across more than 500 esophagectomies [70]. Thoracic epidural analgesia has also shown benefits in improving microcirculation, potentially reducing AL risk by enhancing perfusion at the anastomotic site [71].

Careful pre- and perioperative management is essential for minimizing AL risk, underscoring the need for an individualized approach. Addressing modifiable factors and accounting for each patient’s unique risk profile enables clinicians to implement targeted interventions that optimize postoperative outcomes. Strategies such as minimizing blood transfusions, optimizing operative times, employing goal-directed fluid therapy, and maintaining hemodynamic stability through standardized protocols have demonstrated significant benefits. Additionally, effective pain management and perfusion-enhancing measures, including thoracic epidural analgesia and intraoperative hemodynamic optimization, further reduce ischemic risks at the anastomotic site. These approaches emphasize the importance of a multidisciplinary effort, integrating surgical, anesthetic, and perioperative care teams to achieve optimal outcomes for esophagectomy patients.

Surgical Factors

Surgical technique and intraoperative decisions play pivotal roles in determining AL risk. Data from the Society of Thoracic Surgeons database reveal variations in AL rates across different surgical approaches [72]. While overall anastomotic complication rates are similar between minimally invasive and open esophagectomy, subgroup analysis shows that patients undergoing a minimally invasive Ivor Lewis procedure have twice the rate of nonoperatively managed AL compared to other techniques (8.3% vs. 4.1%; P < 0.001) [72].

Robotic-assisted minimally invasive esophagectomy offers potential advantages in reducing AL rates through enhanced precision and reduced tissue trauma [59]. One study observed a trend toward fewer leaks in robotic esophagectomy compared to standard thoracolaparoscopic approaches (3% vs. 12.5%; P = 0.057), though the difference was not statistically significant [73]. Another study reported an AL rate of 13% among 40 patients undergoing robotic Ivor Lewis esophagectomy with a robotic hand-sewn anastomosis, highlighting the challenges associated with advanced techniques despite their potential benefits [74]. Across studies, AL rates for robotic-assisted esophagectomy range from 3.0% to 24.1% (compared to 7.0% to 25.3% for open esophagectomy), influenced by anastomotic location, surgeon expertise, and procedural variability [75]. These findings emphasize the importance of structured training and surgical experience in optimizing outcomes. Nevertheless, the feasibility and safety of robotic approaches are supported by reports of median operative times around 320 minutes with no conversions to open surgery when performed by experienced surgeons [74].

Van der Sluis et al. prospectively analyzed 100 patients undergoing robotic-assisted Ivor Lewis esophagectomy and found an AL rate of 8%, with low associated mortality (1% at 30 days and 3% at 90 days) [76]. Wu et al. analyzed 2,696 esophagectomy patients and reported a 14% incidence of AL, with no significant differences across surgical approaches (13% open, 14% laparoscopic, 18% robotic; P = 0.123) [77].

Despite these advancements, AL rates remain highly variable, ranging from as low as 3% in some robotic cohorts to over 20% in certain minimally invasive procedures. Most studies report no significant difference in AL incidence between open, minimally invasive, or hybrid approaches, suggesting that surgical technique alone may not be the primary determinant of AL risk [6,78]. Perioperative factors, including blood transfusions, incomplete anastomotic rings, and technical challenges, also contribute to these disparities [26]. While minimally invasive and robotic techniques hold promise in reducing morbidity, substantial expertise is required to minimize complications and optimize outcomes.

The impact of the surgical learning curve on AL risk is well-documented. Studies show a significant decrease in AL and gastric tube necrosis rates as surgical experience increases. AL rates dropped from 18% during the initial 50 cases to 7% in subsequent procedures (P = 0.0457) [79]. Similarly, an analysis of 646 patients undergoing minimally invasive esophagectomy across multiple centers observed a decline in AL incidence from 18.8% during the learning phase to 4.5% after 119 cases (P < 0.001). Notably, 10.1% of ALs were attributed to the learning curve phase, underscoring the critical role of surgical technique and experience in improving outcomes [80].

The choice between transthoracic esophagectomy (TTE) and transhiatal esophagectomy (THE) significantly influences AL rates, with TTE demonstrating potential advantages in reducing leak rates. Studies report a lower AL incidence with TTE (7.2%) compared to THE (13.6%), and meta-analyses of 52 studies involving 5,905 patients (3,389 undergoing TTE and 2,516 undergoing THE) consistently show lower AL rates following TTE (10.6%) compared to THE (16.9%) [81,82]. Ryan et al.'s meta-analysis of 21 studies, including three randomized controlled trials with 7,167 patients, corroborated these findings, identifying higher AL rates after THE (12%) compared to TTE (9.8%) [83]. Schuchert et al. further highlighted that ALs following THE, while comparable in mortality outcomes to those after TTE, are associated with increased morbidity (70% vs. 47%, P = 0.02), longer hospital stays (18 vs. 10 days, P = 0.048), and a higher incidence of anastomotic strictures (57% vs. 19%, P = 0.0001) [84]. Stricture formation often necessitates multiple postoperative dilations, impacting long-term swallowing function and quality of life. The study’s classification system for AL severity underscores the need for standardized assessment to guide management strategies and facilitate consistent comparisons across surgical approaches [84].

The extent and technique of lymph node dissection during esophagectomy significantly influence AL risk, making it a critical factor in surgical planning [34]. Studies have identified lymph node dissection status as an independent risk factor for AL, with a higher incidence observed in patients undergoing 3-field lymph node dissection compared to 2-field dissection. One study reported an AL incidence of 51.1% in the 3-field group versus 26.7% in the 2-field group (P = 0.007) [85]. Retrospective analyses have similarly demonstrated the elevated AL risk associated with 3-field lymph node dissection [86]. A meta-analysis of over 7,000 patients from two randomized controlled trials and 18 observational studies revealed a significantly higher AL risk with 3-field dissection compared to 2-field dissection (RR = 1.26; 95% CI: 1.05-1.52; P = 0.09) [87].

Technical Considerations

Anastomotic techniques play a crucial role in determining leak rates, with no clear consensus on the optimal method. End-to-end configurations generally demonstrate lower leak rates compared to end-to-side techniques, particularly in cervical anastomoses [88]. While hand-sewn anastomoses, often used in cervical cases, typically employ single-layer continuous sutures, two-layered techniques may further reduce leaks [89]. Notably, a side-to-side stapled cervical esophagogastric anastomosis has shown significant advancements, reducing leak rates from 10-15% (manual sutures) to 2.7%, shortening hospital stays, and improving patient satisfaction, underscoring its potential as a superior option in certain settings [90,91].

In intrathoracic anastomoses, stapling is more common. While linear-stapled techniques are sometimes considered superior to hand-sewn methods, studies have not consistently demonstrated significant differences in leak rates or outcomes [37]. Similarly, comparisons among circular mechanical, linear mechanical, and hand-sewn techniques reveal comparable results, suggesting no single approach is definitively superior [92,93]. Cervical anastomoses, in contrast, often employ semi-mechanical methods that combine linear staplers and hand-sewing for side-to-side esophagogastrostomy or fully mechanical techniques using staplers alone to reduce operative time and strictures [94]. Regardless of the anastomotic method, principles such as tension-free construction and coverage with the omentum remain critical in reducing complications and improving outcomes.

Omental reinforcement has emerged as an effective strategy to reduce AL rates (Video 3). The omentum’s ability to form adhesions and seal microscopic leaks enhances its protective role in both cervical and intrathoracic anastomoses [95]. Sepesi et al. demonstrated a significant reduction in AL rates with omental reinforcement in patients undergoing esophagectomy with thoracic anastomosis (4.7% vs. 10.5%; P = 0.04) [13].

**Video 3:** Omental flap and conduit suspension for anastomotic support and perfusion optimization. This video is from the author's own research and demonstrates the use of an omental flap for conduit suspension in esophageal surgery, highlighting its role in reinforcing the anastomosis and improving vascular perfusion. The omental flap is mobilized to support the gastric conduit, helping to reduce tension, enhance healing, and minimize the risk of anastomotic complications such as leaks or strictures. This technique provides a well-vascularized, protective barrier around the anastomotic site, optimizing postoperative outcomes.

Adding further evidence, Bhat et al. conducted a prospective randomized study on 238 patients undergoing esophagogastrectomy for esophageal carcinoma, comparing outcomes with and without pedicled omental wrapping around the esophagogastric anastomosis [96]. Patients who received omental wrapping exhibited a significantly lower AL rate (3.09%) compared to those without (14.43%; P = 0.005). Importantly, the study reported no complications associated with the omental graft technique and no significant difference in mortality between the two approaches [96]. These findings highlight pedicled omental reinforcement as an effective strategy for protecting the anastomotic suture line and significantly reducing postoperative leak rates and associated morbidity and mortality.

A prospective randomized study by Dai et al. further supports the utility of omental reinforcement [97]. Involving 255 patients undergoing esophagogastrectomy, the study compared outcomes between patients receiving pedicled omental flap wrapping and those undergoing a standard stapled anastomosis [97]. Omental reinforcement significantly reduced the incidence of AL (1% vs. 6%; P < 0.05) and strictures (6% vs. 16%; P < 0.05), underscoring its effectiveness in minimizing postoperative complications [97]. Neither the Bhat nor the Dai trial demonstrated significant differences in postoperative complication rates, hospital mortality, length of stay, or incidence of anastomotic strictures.

Zheng et al. conducted a prospective study involving 184 patients undergoing radical esophagectomy with three-field lymphadenectomy, randomized into groups with and without omental reinforcement [98]. The omental reinforcement group demonstrated a significantly lower incidence of AL (3.3%) compared to the non-reinforcement group (9.8%; P < 0.05). Importantly, no lethal leaks occurred in the reinforcement group, whereas two patients in the non-reinforcement group succumbed to empyema and mediastinitis. Anastomotic stricture rates were comparable between the groups (4.3% vs. 2.2%; P > 0.05). Patients who underwent omental reinforcement also had significantly shorter hospital stays (P < 0.05), with no notable differences in overall mortality, tumor recurrence, or metastasis rates (P > 0.05) [98].

Further support comes from Yoshida et al., who analyzed 338 patients undergoing McKeown esophagectomy with gastric conduit reconstruction, comparing outcomes with and without omental reinforcement [99]. After propensity score matching, omental reinforcement significantly reduced AL rates (P = 0.016) and surgical site infections (P = 0.041). Independent risk factors for AL included the absence of omental reinforcement (OR 2.55; 95% CI: 1.234-5.285; P = 0.0088) and younger age (OR 1.06; 95% CI: 1.012-1.102; P = 0.011) [99].

Similarly, Yuan et al.’s Cochrane review of three RCTs found that while omental reinforcement did not significantly affect hospital mortality (RR 1.28; 95% CI: 0.49-3.39) or long-term survival, it significantly reduced postoperative AL incidence (RR 0.25; 95% CI: 0.11-0.55) [100]. However, other studies, such as one by Lu et al., observed no significant differences in AL rates (16% vs. 13%; P = 0.512), morbidity, or mortality with omental reinforcement in patients undergoing minimally invasive esophagectomy after nCRT, indicating that its efficacy may depend on patient populations and perioperative strategies [101].

Collectively, these findings establish omental reinforcement as a promising strategy for preventing AL, particularly in specific surgical and clinical contexts. However, its effectiveness may vary depending on patient characteristics, surgical techniques, and perioperative management strategies. These observations emphasize the importance of individualized surgical planning and further research to refine its role in optimizing outcomes across diverse clinical settings.

Conduit width also plays a critical role in anastomotic integrity. Narrower conduits (3-4 cm in diameter) are associated with higher AL rates (27.6%) compared to wider conduits (6 cm diameter) with a leak rate of 6.1%, likely due to compromised submucosal perfusion in narrower conduits [102]. The vascular supply to the uppermost portion of the gastric conduit is particularly delicate, making wider conduits less prone to ischemic complications and AL [14]. However, creating wider conduits may compromise surgical margins in patients with proximal tumors, potentially increasing the risk of tumor recurrence, or contribute to conduit stasis and retention, increasing the risk of aspiration. This highlights the need for careful surgical planning to balance oncological objectives, long-term outcomes, and anastomotic integrity.

Regardless of the technique, meticulous attention to technical details is crucial in preserving anastomotic integrity. Avoiding excessive traction, twisting, or compression of the conduit and ensuring complete donuts in circular mechanical anastomoses are essential. Caution is also necessary to prevent over-reliance on mechanical devices such as staplers, which can contribute to complications if improperly used.

Preemptive endoluminal vacuum therapy (EVT) is an innovative approach aimed at reducing AL rates and postoperative morbidity following esophagectomy. In a study involving 19 patients undergoing minimally invasive esophagectomy, EVT was applied immediately after esophagogastrostomy and removed routinely after a median of 5 days. Among the patients, 63% were high-risk cases with severe comorbidities. AL occurred in only one minor case (5%), which healed following a second course of EVT. Anastomotic healing was uneventful in 95% of cases, with no EVT-related adverse events except for one instance of early proximal dislodgement. The 30-day mortality rate was 0%, and the median comprehensive complication index was 20.9 [103]. These findings suggest that preemptive EVT may be an effective strategy for improving outcomes in esophagectomy patients.

Another case series evaluated the preemptive use of EVT for treating anastomotic ischemia following esophagectomy. Eight patients underwent intraluminal EVT, achieving complete mucosal recovery in six patients (75%), while two developed small anastomotic leaks that resolved with continued EVT. The median treatment duration was 16 days (range 6-35), with a median of five sponge changes (range 2-11). No EVT-related complications were observed, although three patients developed anastomotic stenoses requiring endoscopic dilation [104]. This study suggests that early EVT may improve clinical outcomes and reduce infection risk in cases of anastomotic ischemia, highlighting the need for further research to refine its indications and identify optimal candidates.

The development of AL results from a complex interplay of patient-specific factors, anatomical and physiological challenges, surgical techniques, and perioperative management strategies. A comprehensive understanding of these interconnected risk factors enables clinicians to implement tailored interventions that minimize the likelihood of AL and optimize outcomes for esophagectomy patients. Addressing modifiable elements such as improving conduit perfusion, employing advanced imaging technologies, and adhering to standardized perioperative protocols has shown significant potential in reducing AL incidence and associated morbidity.

Preemptive strategies such as omental reinforcement and endoluminal vacuum therapy, along with technical refinements in surgical techniques, highlight promising avenues for reducing AL rates. Minimally invasive and robotic techniques offer unique advantages, including reduced tissue trauma and enhanced precision. However, their success is highly dependent on surgical expertise, technical proficiency, and thoughtful patient selection. The surgical learning curve remains a crucial factor, with evidence demonstrating significant reductions in AL rates as surgical teams gain experience and refine their techniques. A multidisciplinary approach that combines personalized patient assessment, evidence-based practices, and innovative technologies is essential to addressing the multifaceted nature of AL.

Diagnosis of anastomotic leak

The diagnosis of AL remains a challenging and critical aspect of postoperative care, with no universal consensus on the optimal diagnostic process, timing, or choice of modalities. Accurate and timely detection of AL is essential to mitigate complications, reduce clinical burden, and improve patient outcomes. A comprehensive diagnostic approach incorporates clinical assessment, biochemical markers, imaging studies, and endoscopic evaluations, tailored to the patient’s presentation and risk factors.

Clinical Presentation

The clinical manifestations of AL are highly variable, ranging from asymptomatic cases to life-threatening sepsis. Factors influencing presentation include the anatomical location of the anastomosis, defect size, leak containment, and drainage efficiency. Early signs may be subtle, such as fever or wound erythema in cervical anastomoses. Tachycardia, frequently manifesting as atrial fibrillation, is often the earliest indicator [105].

In cervical leaks, erythema or induration at the neck incision, discharge of saliva-like fluid, pus, or air, and occasionally infection extending into the thorax are common. Mediastinal abscesses, pleural empyema, and tracheoesophageal fistulae are more prevalent after transthoracic esophagectomy due to extensive pleural dissection [106]. In contrast, the transhiatal approach, which spares the parietal pleura, may limit the spread of thoracic infection. However, transthoracic procedures can exacerbate complications due to negative intrathoracic pressure facilitating infection dissemination, leading to empyema, bronchopneumonia, mediastinitis, respiratory failure, and, in severe cases, multi-organ failure [107]. Signs such as the presence of saliva or gastric contents in surgical drains and persistent coughing during swallowing also suggest AL.

Biochemical Markers

Biochemical analyses complement clinical evaluation, with inflammatory markers such as C-reactive protein (CRP), procalcitonin, and white blood cell (WBC) counts serving as early indicators of AL. Among these, CRP measured on postoperative day (POD) 3 has shown strong diagnostic utility [108]. Elevated CRP on POD 3 is highly associated with AL, with a pooled cutoff of 17.6 mg/dL yielding 74% sensitivity and a POD 5 cutoff of 13.2 mg/dL achieving 86% sensitivity [109,110]. A retrospective study reported that a POD 3 CRP cutoff of 19.0 mg/dL provided 100% sensitivity, making it a reliable exclusionary tool for patients below this threshold [111]. However, CRP’s limited specificity prevents its use as a standalone diagnostic method.

Trends in CRP levels offer additional insights. Rising CRP levels between POD 3 and POD 5 correlate with AL, while a downward trend generally rules out leaks [112]. The CRP-to-albumin ratio is another promising tool, with Shao et al. reporting a sensitivity of 80% and specificity of 98.8% using a classification and regression tree (CART) model [113].

Drain amylase levels provide valuable diagnostic insights into AL. Elevated amylase levels in surgical drains are indicative of salivary contamination and potential leakage [114]. On POD 3, amylase cutoff values ranging from 160 to 554 IU/L demonstrate sensitivities of 35.7%-75% and specificities of 83.8%-98% [112]. By POD 4, cutoffs between 31 and 250 IU/L yield improved sensitivities of 75%-100% and specificities of 52%-95.5% [115]. Additionally, low pleural drain amylase levels (<38 IU/L) can help reassure clinicians in cases with low suspicion of AL and normal inflammatory markers. However, the diagnostic utility of drain amylase levels in distinguishing cervical from intrathoracic leaks remains poorly understood and warrants further investigation.

Tang et al. retrospectively reviewed 414 patients undergoing esophagectomy with intrathoracic anastomosis to evaluate the role of prophylactic perianastomotic drains [116]. Among the 112 patients with prophylactic drains, leaks were identified early based on drain output and confirmed at the bedside using methylene blue, avoiding delays associated with imaging. In contrast, eight patients in the no-drain group required additional drains for leak management, with significantly longer leak resolution times (mean 23.4 vs. 80.7 days; P < 0.05). Prophylactic drain placement also facilitated faster diagnosis and recovery, enabling an earlier return to oral intake (mean 32.2 vs. 98 days; P < 0.05) and reducing the need for additional interventions [116]. While these findings are promising, routine use of prophylactic drains in esophagectomy has been established as a current standard of care.

Diagnostic Imaging

Imaging plays a pivotal role in AL detection, with modalities such as fluoroscopic contrast swallow studies and computed tomography (CT) utilized based on clinical context. Contrast swallow studies, such as esophagography, remain a widely used and cost-effective initial diagnostic tool. These studies provide valuable information about the contour of the replacement conduit, its emptying capacity, and pyloric patency. However, their sensitivity in detecting AL varies significantly, with reported rates ranging between 33% and 52% [117]. Specific findings such as contrast extravasation confirm leaks but can yield false negatives, particularly for cervical anastomoses [118]. Honing et al. advocated for on-demand contrast imaging based on clinical suspicion, identifying sepsis, leukocytosis, and fever as key predictors [119].

CT imaging remains the cornerstone for diagnosing AL, offering the ability to evaluate the neck, thorax, and abdomen simultaneously [120]. It effectively detects fluid collections, air pockets, and esophageal wall defects while identifying complications such as pulmonary effusion, abscesses, and pneumonia [27]. Despite its utility, the diagnostic accuracy of CT varies due to the absence of standardized interpretation criteria [121]. Findings such as mediastinal air or fluid, while suggestive, are nonspecific and can also appear following uncomplicated esophagectomy [118]. CT scans demonstrate higher sensitivity than contrast swallow studies in cases of clinical suspicion, and sensitivity increases in the presence of mediastinal gas, though this improvement comes at the cost of reduced specificity [118,122]. Both CT with oral contrast and esophagography generally exhibit comparable specificity. Combining CT findings with additional diagnostic tools, such as drain amylase levels, enhances sensitivity, achieving a specificity of 95.8% in some studies [123].

Innovations such as measuring air bubble density in the mediastinal space have shown promise, with sensitivity of 86.4% and specificity of 95.8%, though further validation is required to confirm their clinical utility [124]. Other advancements, such as CT-based scoring systems that integrate findings like mediastinal fluid and esophagogastric wall discontinuity, have further improved diagnostic accuracy, achieving sensitivity and specificity rates of 80% and 84%, respectively. A score of ≥2 correlated well with systematic assessments performed by two independent radiologists, highlighting the potential for standardized scoring to improve AL detection [121].

The combination of CT and other modalities is highlighted by Strauss et al., who reported that CT scans performed on POD 3 had limited diagnostic value, but combining CT with contrast swallow studies at POD 7 improved sensitivity and negative predictive value to 95.8% [125]. Endoscopy clarified inconclusive CT findings, confirming AL in only one of 16 cases [125]. This multimodal approach allows for more accurate assessment before reintroducing oral feeding.

Endoscopy

Upper gastrointestinal endoscopy is highly sensitive and specific for diagnosing AL, allowing direct visualization of the anastomosis, identification of defects, and therapeutic interventions for leak management. Sensitivity and specificity can reach 95%, though cervical leaks demonstrate lower sensitivity (56%) [126,127]. The role of routine endoscopy remains debated. While some studies support its utility in detecting AL [128], others find it ineffective in predicting future leak development [129]. Page et al. demonstrated that endoscopy achieved 100% accuracy in diagnosing AL, underscoring its reliability when clinical suspicion is high [130].

Despite its advantages, endoscopy has limitations, including its inability to assess perianastomotic conditions and the need for sedation, which increases risks in critically ill patients.

Timing of Diagnosis

The timing of diagnostic procedures is critical for minimizing complications and improving outcomes. However, the optimal timing remains a debated issue in managing AL [131]. Early detection is essential for preventing severe complications, reducing hospital stays, and improving overall prognosis. An on-demand diagnostic approach, guided by clinical signs and inflammatory markers, is commonly utilized, reserving imaging and endoscopic investigations for patients with evident clinical or biochemical abnormalities [114]. However, this strategy has limitations, as it relies on clinicians recognizing subtle signs of deterioration, potentially delaying diagnosis.

Routine endoscopy after esophagectomy has been proposed to detect AL in asymptomatic patients, though its utility remains controversial [6]. Some studies suggest that routine endoscopy between POD 7 and 14 may predict leaks by identifying early mucosal degeneration, such as ischemia or necrosis [128]. Similarly, Fujiwara et al. demonstrated the utility of early endoscopy on POD 1 in 153 patients undergoing esophageal reconstruction, identifying mucosal color changes (MCC) in 36 patients as a marker of local circulatory failure in the gastric graft [132]. Patients with MCC had significantly higher rates of poor anastomotic healing one week later (55.6%) compared to those with normal findings (2.6%; P < 0.001) [132]. These findings highlight early endoscopy as a potential predictive tool for identifying compromised healing.

Despite these findings, routine radiological contrast swallow (RRCS) studies on POD 5 have shown limited diagnostic value. Elliott et al. demonstrated that early esophagrams have a sensitivity of only 51% for AL, with missed leaks leading to more severe outcomes [19]. Of the 55 patients ultimately diagnosed with AL, 27 (49%) initially had normal esophagram findings, which were performed on average on POD 6. Patients with normal esophagram results were significantly more likely to develop uncontained leaks compared to those with abnormal findings (81% vs. 29%; P < 0.01) [19]. Analysis of 328 esophagectomy patients revealed an AL rate of 15%, with 23% diagnosed before RRCS, 69% after an unremarkable RRCS, and only 8% detected during RRCS. The sensitivity of RRCS was only 16%, with most leaks occurring between POD 1 and 19. CT proved more effective, identifying 41% of AL cases, leading to recommendations for CT with oral contrast and endoscopy when AL is suspected instead of routine RRCS [133]. Similarly, routine anastomotic checks between PODs 5 and 7 were shown to have limited value, with only 3 of 11 pathological findings leading to AL. Additionally, seven of 83 patients developed AL despite negative routine checks, underscoring the limited sensitivity of routine imaging in asymptomatic patients [129].

These findings collectively highlight the limitations of routine diagnostic imaging and advocate for an on-demand approach. This strategy, supplemented by advanced diagnostic modalities such as CT imaging and endoscopy, provides a more sensitive and tailored method for detecting AL in patients with clinical suspicion of leaks.

The diagnosis of AL after esophagectomy is a complex and multifaceted challenge that requires a tailored, multimodal approach to ensure timely and accurate detection. While clinical assessment remains the cornerstone of diagnosis, its variable presentation necessitates the integration of biochemical markers, imaging studies, and endoscopic evaluations. Prophylactic drain placement and on-demand diagnostic strategies further facilitate early identification and management of leaks, minimizing complications and expediting recovery. Given the limited sensitivity of routine imaging and endoscopy in asymptomatic patients, targeted diagnostics guided by clinical suspicion and inflammatory markers remain paramount in optimizing patient outcomes.

## Conclusions

AL remains one of the most consequential complications following esophagectomy, significantly impacting both short-term morbidity and long-term oncologic outcomes. Despite advancements in surgical techniques, optimization of perioperative management, and improvements in diagnostic modalities, AL continues to pose a substantial challenge due to its multifactorial nature. A comprehensive understanding of its pathophysiology, risk factors, and evolving management strategies is crucial for improving patient outcomes. Emerging technologies such as intraoperative fluorescence imaging, endoluminal vacuum therapy, and enhanced perfusion assessment tools have shown promise in mitigating ischemic-related leaks. Additionally, multidisciplinary perioperative care, including nutritional optimization, goal-directed fluid therapy, and standardized hemodynamic protocols, has contributed to improved surgical recovery and reduced complication rates. However, variability in surgical approaches, anastomotic techniques, and postoperative management underscores the need for individualized strategies tailored to patient- and procedure-specific risks.

The timely and accurate diagnosis of AL remains critical for reducing mortality and morbidity. While traditional diagnostic approaches such as contrast esophagography and CT remain widely used, their limitations in sensitivity necessitate a multimodal strategy. The integration of inflammatory markers, perianastomotic drain monitoring, and advanced imaging techniques has enhanced early leak detection, allowing for timely intervention. Additionally, endoscopic evaluation plays an increasingly important role, not only in diagnosing AL but also in guiding minimally invasive therapeutic interventions such as stenting and vacuum-assisted closure. Future advancements in real-time perfusion assessment, predictive algorithms, and tailored surgical techniques will further refine strategies for AL prevention and management. As the field continues to evolve, optimizing perioperative protocols and leveraging technological innovations will be instrumental in improving patient outcomes and minimizing the burden of this formidable complication in esophageal surgery.
